# Systemic and Nonrenal Adverse Effects Occurring in Renal Transplant Patients Treated with mTOR Inhibitors

**DOI:** 10.1155/2013/403280

**Published:** 2013-09-19

**Authors:** Gianluigi Zaza, Paola Tomei, Paolo Ria, Simona Granata, Luigino Boschiero, Antonio Lupo

**Affiliations:** ^1^Renal Unit, Department of Medicine, University Hospital of Verona, Piazzale Stefani 1, 37126 Verona, Italy; ^2^First Surgical Clinic, Kidney Transplantation Center, University Hospital of Verona, Piazzale Stefani 1, 37126 Verona, Italy

## Abstract

The mammalian target of rapamycin inhibitors (mTOR-I), sirolimus and everolimus, are immunosuppressive drugs largely used in renal transplantation. The main mechanism of action of these drugs is the inhibition of the mammalian target of rapamycin (mTOR), a regulatory protein kinase involved in lymphocyte proliferation. Additionally, the inhibition of the crosstalk among mTORC1, mTORC2, and PI3K confers the antineoplastic activities of these drugs. Because of their specific pharmacological characteristics and their relative lack of nephrotoxicity, these inhibitors are valid option to calcineurine inhibitors (CNIs) for maintenance immunosuppression in renal transplant recipients with chronic allograft nephropathy. However, as other immunosuppressive drugs, mTOR-I may induce the development of several adverse effects that need to be early recognized and treated to avoid severe illness in renal transplant patients. In particular, mTOR-I may induce systemic nonnephrological side effects including pulmonary toxicity, hematological disorders, dysmetabolism, lymphedema, stomatitis, cutaneous adverse effects, and fertility/gonadic toxicity. Although most of the adverse effects are dose related, it is extremely important for clinicians to early recognize them in order to reduce dosage or discontinue mTOR-I treatment avoiding the onset and development of severe clinical complications.

## 1. Role and Biological Function of mTOR Inhibitors (mTOR-I)

 The mammalian target of rapamycin inhibitors (mTOR-I), sirolimus and everolimus, are agents with many immunosuppressive and anti-cancer properties [1]. 

The main mechanism of action of these drugs is the inhibition of mammalian target of rapamycin (mTOR). mTOR is a regulatory protein kinase involved in lymphocyte proliferation, developmental processes such as neurologic and muscle generation, and tumor cell growth. Sirolimus (SRL; Rapamune, Wyeth Pharmaceuticals, New York City, NY, USA) was the first mTOR inhibitor (mTOR-I) approved for use in renal transplant recipients. It binds to the immunophilin FK binding protein-12 (FKBP-12). Everolimus (EVR), marketed as Certican, was approved lately, and it is structurally similar to SLR except for the addition of an extra hydroxyethyl group at position 40 [2]. Whereas the Tacrolimus (TAC)/FKBP-12 complex inhibits calcineurin-induced transcription of interleukin-2 (IL-2), the SRL/FKBP-12 and EVR/FKBP-12 complexes both bind directly to mTOR, halting T-cell progression from the G1 to the S phase of cell cycle, leading to inhibition of IL-2-induced protein synthesis and cellular proliferation [3]. 

Because of their specific pharmacological characteristics, mTOR-I are highly effective in renal transplantation, and thanks to their relative lack of nephrotoxicity, these inhibitors are a valid option to calcineurin inhibitors (CNIs) for maintenance of immune suppression in renal transplant recipients with chronic allograft nephropathy [4–6]. However, as reported by recent studies [7, 8], it seems clear that time and drug dosage may have a primary role in the development of drug-related adverse effects and clinical complications. 

Additionally, the inhibition of the crosstalk among mTORC1, mTORC2, and phosphatidylinositol-3 kinase (PI3K) confers the antineoplastic activities of these drugs [9]. EVR received Food and Drug Administration (FDA) approval in 2009 for renal cancer carcinoma (RCC) and successively for tuberous sclerosis and pancreatic neuroendocrine tumors [10, 11]. The anticancer efficacy of mTOR-I seems to be limited to their cytostatic and no cytotoxic activities, so the clinical effect is stabilization rather than regression. Therefore these drugs are extremely useful for the immunosuppressive treatment of patients developing posttransplant neoplasias [9].

The mechanism of antitumor activity is also correlated to the upregulation of adhesion molecules and to a switch to less invasive phenotype of tumoral cells. Moreover, the inhibition of angiogenesis is due to the reduction of vascular endothelial growth factor (VEGF) production and decreased endothelial sensitivity to such growth factor [12–14].

Moreover, mTOR-I may reduce the incidence of several comorbidities associated with transplantation and chronic kidney disease including atherosclerosis [15] and complications correlated to polycystic kidney disease [16, 17].

Although the clinical utility of this drug category is clear, as other immunosuppressive drugs, mTOR-I may induce the development of several adverse effects (Table 1) that need to be early recognized and treated to avoid severe illness in renal transplant patients.

However, although the majority of the mTOR-I clinical trials have been performed in renal transplant patients using SRL, it seems reasonable that EVR may induce similar adverse effects. Sánchez-Fructuoso et al. have recently reported no difference in the rate of drug discontinuation for adverse effects between EVR- and SRL-treated patients [18]. In addition, it is conceivable that most results from initial clinical trials using SRL can not be compared with those obtained by more recent studies using EVR mainly because of the different dosages used, dissimilar trough levels reached and drug combinations proposed. Nevertheless, randomized clinical trials are necessary to better address this important clinical research topic.

In this review we focus our attention only on the main nonrenal adverse effects/toxicities occurring in renal transplant patients treated with both mTOR inhibitors. 

## 2. Pulmonary Toxicity

Pulmonary adverse effects/toxicities are highly frequent in renal transplant recipients treated with mTOR-I. Numerous clinical studies have reported a frequency of this complication of 2–11% with the onset of symptoms between 1 and 51 months after the initiation of SRL or EVR therapy [19–22]. 

MTOR-I-associated pneumonitis has heterogeneous clinical manifestations and may begin with fever, fatigue, coughing and dyspnoea, and nonspecific signs and symptoms, which do not facilitate diagnosis [23]. 

Several distinct types of pulmonary damage have been recognized, including lymphocytic interstitial pneumonitis, lymphocytic alveolitis, bronchiolitis obliterans with organizing pneumonia, focal pulmonary fibrosis, or a combination thereof [24, 25]. Diffuse alveolar hemorrhage has been reported following the use of both SRL and EVR [26, 27].

The etiopathogenic mechanism of mTOR-I-associated pulmonary toxicity is still unclear, and several *in vivo* and *in vitro* studies have tried to define the biological machinery associated with this heterogeneous clinical condition. 

A cell-mediated autoimmune response may have a pivotal role when cryptic pulmonary antigens are exposed, and this causes lymphocytic alveolitis and interstitial pneumonitis. T-cell-mediated, delayed-type hypersensitivity may be another pathogenic mechanism [19]. Additionally, Ussavarungsi et al. have recently reported that SRL may induce granulomatous interstitial inflammation which suggests a role of T-cell-mediated hypersensitivity reaction to circulating antigens or immune complexes in the lungs [28]. T-cell lymphocytes produce IL-2 and IFN-gamma which stimulate alveolar macrophages and also produce TNF-alpha and IL-1. Activated macrophages secrete several chemokines such as monocyte chemoattractant protein-1 (MCP-1) and macrophage inflammatory protein 1 alpha (MIP-1*α*) and transform into epithelioid cells and multinucleated cells contributing to cellular infiltration and granuloma formation [29]. Additionally, the existence of a dose-dependent effect was strongly suggested because this disease has been observed particularly in kidney transplant patients receiving high doses of SRL. 

Therefore, to assume a possible correlation between pulmonary disease and mTOR-I administration, patients should present the following conditions:

1. exposure to mTOR-I preceding the onset of pulmonary symptoms;
2. exclusion of infection or alternative pulmonary disease, including toxicity due to other drugs, such as azathioprine, beta-blockers, fibrates, sulfamethoxazole, and trimethoprim;
3. resolution after mTOR-I discontinuation or minimization;
4. presentation of a lymphocytic alveolar cellular profile and pathological findings, although nonspecific, consistent with drug-induced pulmonary toxicity.

Then, to achieve a correct diagnosis, radiographic tests, computer tomography (CT) and bronchoalveolar lavage (BAL), even whether often unspecific, are extremely useful. CT images generally describe ground glass opacities and/or peripheral interstitial infiltrates, while BAL generally shows an increased number of CD4+ cells with mast cells and eosinophils [19]. It is unquestionable that, when possible, BAL should be performed to rule out infectious processes in these immunocompromised patients. Then, pulmonary function tests, demonstrating a reduction of the diffusing capacity of carbon monoxide, are less helpful, with this condition being in overlap with several other clinical features [30, 31].

In most cases, it is also necessary to early perform a lung biopsy. Usually, the lung biopsy pattern may include several histological features such as the intra-alveolar nonnecrotizing epitheloid granuloma, lymphocytic interstitial inflammation, and a focal pattern of organizing pneumonia [32]. 

The treatment of these pulmonary forms is variable, and it needs a multidisciplinary approach (e.g., pneumologists, infectiologists, and nephrologists) and a combined use of several drugs (e.g., antibiotics, corticosteroids, and immunosuppressive drugs). Nevertheless, as reported by Weiner et al., the treatment of choice of these complications results generally in drug withdrawal [33, 34]. However, in some cases, mTOR-I dose reduction could be sufficient to control symptoms and avoid disease progression/complication. As described by White et al. the same strategies have been adopted in patients affected by advanced renal cell carcinoma chronically treated with EVR [30]. Additionally, a published overview of cases of mTOR inhibitor-related noninfectious pneumonitis in liver transplant recipients (104 treated with SRL and 9 with EVR) confirms this therapeutic strategy. In fact it was reported that, after diagnosis of mTOR-I pulmonary adverse effects, SRL was withdrawn in 87/104 patients, with complete resolution in 82 patients, partial resolution in 1 patient, and 4 deaths. Of the 13/104 patients who continued SRL at a reduced dose, 8 showed complete resolution and the remaining 5 either had persistent symptoms or relapsed. All five patients had complete resolution on drug discontinuation. Among the nine patients treated by EVR, drug was withdrawn in eight patients and continued at a reduced dose in one patient. All experienced complete recovery [35]. 

Concerning corticosteroids, it remains unclear whether these drugs may be useful to treat mTOR-I-related pulmonary complications [31]. No studies have adequately analyzed the effect of additional/different therapies for the treatment of pulmonary adverse effects/toxicities occurring in renal transplant recipients.

Therefore, in conclusion, all clinicians in charge of transplanted patients should be aware of this new entity of mTOR-I-associated pneumonitis as an alternative to the diagnosis of an opportunistic infection. Indeed, discontinuation or dose reduction of mTOR-I led in most of the cases to the complete and lasting resolution of symptoms.

## 3. Hematopoietic Adverse Effects

Hematopoietic adverse effects and bone marrow toxicity often occur after renal transplantation in patients undergoing mTOR inhibition. In particular, mTOR-I-treated patients develop anemia, leukopenia, and thrombocytopenia [36, 37].

### 3.1. Anemia

Anemia is common after kidney transplantation and has been recognized as a late complication of transplantation [38, 39]. It has been linked to multiple factors including poor allograft function, acute and chronic rejection, iron deficiency, viral infections, hemolytic uremic syndrome, treatment with angiotensin converting-enzyme inhibitors (ACEIs) or angiotensin receptor blockers (ARBs) and immunosuppressive drugs [40–43]. 

Initial phases 1 and 2 dose escalation studies found a slower recovery of postoperative hemoglobin (Hb) levels in SRL-Cyclosporin A (CsA)/prednisone-treated compared with CsA/prednisone-treated renal transplant recipients [44]. Groth et al. reported an approximately 35% incidence of early posttransplant anemia in SRL-treated patients versus 25% in a CsA-treated group [45]. Kreis et al. observed a 43% incidence of anemia with SRL compared with 32% in the CsA arm in studies in which both treatment arms received either azathioprine or mycophenolate mofetil (MMF) [46]. 

A dose relationship between SRL and anemia development was documented in phase 3 trials comparing SRL 2 mg/day with SRL 5 mg/day administered along with CsA and corticosteroids (24 versus 35%, resp.) [46]. A second phase 3 trial comparing SRL/CsA/corticosteroids with placebo/CsA/corticosteroids found a 36% incidence of anemia (SRL 2 mg/day) versus 56% (SRL 5 mg/day) versus 16% (placebo) [47].

In a study comparing SRL/MMF/prednisone with tacrolimus/MMF/prednisone, mean Hb was again significantly lower in SRL-treated patients at 1 month [48]. Friend et al. found that long-term use of SRL/prednisone resulted in a lower prevalence of anemia compared with SRL/CsA/prednisone, despite higher SRL concentrations [49]. 

Augustine et al. comparing CNI/SRL with CNI/MMF therapy in kidney and kidney-pancreas transplant recipients, found a higher prevalence of anemia in the SRL versus MMF group at 6 months (57 versus 41%, resp.) and 12 months (57 versus 31%, resp.) [36]. These results were confirmed by another study [50]. 

Multiple conversion studies, then, have shown that switching from CNI to SRL in long-term renal transplant patients results in a significant increased degree of anemia, suggesting but not proving a direct causative role. Thaunat et al. reported a decrease of Hb (mean decrease of 2.5 g per 100 mL) in 86.9% of patients switched from CNI-based immunosuppression to SRL-based immunosuppression for chronic allograft nephropathy/chronic CNI nephrotoxicity [51]. Similar results were obtained by Maiorano et al. [52] and Diekmann et al. [53]. The CONVERT trial found that anemia was reported in 37.4% of patients converted to SRL versus 18.7% of patients continued on CNI [54].

Also EVR has been associated with anemia [55, 56]. In the B251 study were reported significantly more anemia-related adverse events at 36 months for EVR 1.5 mg/day and 3 mg/day compared with MMF (32.1% and 39.2% versus 21.4%, resp.) [57].

Various mechanisms have been proposed for mTOR-I-induced anemia, but the complete molecular/biological machinery involved is not fully understood. In patients treated with both SRL and EVR, anemia seems mainly to be due to the antiproliferative effect of the drug on bone marrow progenitor cells and a possible direct impact on iron homeostasis [58]. However, as reported by Thaunat et al. in 2005, there is a close relationship between chronic inflammatory status and mTOR-I-related anemia [51]. Additionally, the same group has recently suggested that SRL might trigger a destabilization of the inflammatory cytokine balance in transplanted patients that promotes a paradoxical inflammatory response with mild stochastic clinical symptoms in the week following drug introduction, explaining partially SRL-associated anemia with low serum iron levels and microinflammation [59]. Similarly, Sánchez Fructuoso et al.[55] reported that after conversion from CNI to EVR there is a significant development of anemia associated with low serum iron levels and microinflammation.

Therefore, based on the multifactorial reasons of the mTOR-I-related anemia, it is now clear that physicians should adopt a rigorous approach to evaluate and treat the anemia in these patients. Transfusion is uncommonly required in pre-, peri-, and posttransplant courses; if performed, leukocyte filtration should be used to decrease the risk of allosensitization and transmission of viral infection (including CMV). The success of EPO-stimulating agents (ESAs) in increasing hemoglobin levels may be related to the cause of anemia and clinical reasons for anemia correction [38, 39]. The data suggest that ESA treatment soon after transplantation shortens the time of hemoglobin correction [60, 61]. Then, iron repletion is important, as even patients with suboptimal erythropoietin levels can correct anemia with adequate iron. Iron deficiency is common in the peritransplant period and treatment decreases the incidence of anemia at six months [62, 63]. 

### 3.2. Leukopenia and Thrombocytopenia

Since the early clinical trials with SRL in transplantation in 1996, it emerged that leukopenia and thrombocytopenia were amongst the most common adverse effects of the mTOR-I. These adverse effects were the most likely reasons for the intervention with mTOR-I to be terminated [44, 45, 64]. During the first 2 months of treatment, dose-dependent thrombocytopenia and leukopenia may occur in approximately 20% of renal transplant recipients [65]. 

Groth et al. reported in a randomized multicentre study [45] including first cadaveric renal allograft recipients randomized to CsA (*n* = 42) or SRL (*n* = 41) in association with corticosteroids and azathioprine that thrombocytopenia and leucopenia were striking adverse effects of SRL. However, these abnormalities improved 2 months after transplantation when the SRL target trough level was lowered from 30 to 15 ng/mL.

In another study from 14 European centres including 78 first cadaveric renal allograft recipients randomized to receive SRL (*n* = 40) or CsA (*n* = 38), a significant higher rate of thrombocytopenia has been reported in the SRL-treated group of patients compared to CNI-treated control (45% versus 8%) [46]. All patients received corticosteroids and MMF 2 g/day. SRL doses were adjusted to achieve steady-state trough levels of approximately 30 ng/mL for 2 months and 15 ng/mL thereafter. 

Also EVR has been associated with both leucopenia and thrombocytopenia. As reported by Kovarik et al. leukocytopenia occurred in 11–19% of patients, while the incidence of thrombocytopenia was 10–17% of patients chronically treated with EVR [66].

One hypothesis to explain the myelosuppressive effects is based on *in vitro* findings that mTOR-I potentiate platelets destruction via agonist-induced aggregation. mTOR-I-treated platelets display increased sensitivity to adenosine diphosphate and/or thrombin receptor agonist peptide, resulting in augmented platelet aggregation and granule secretion [67]. An alternate hypothesis is that the myelosuppressive property of SRL is due to the inhibition of the signal transduction via the gp130 [beta] chain shared by a variety of cytokine receptors, including interleukin-11 [68], granulocyte colony stimulation factor, and erythropoietin, which stimulate the production of platelets, leukocytes, and erythrocytes, respectively [69].

Drug-induced thrombocytopenia and/or leukopenia are generally self-limited toxicities. Only 7% of patients require SRL dose reduction and 4% temporary drug withdrawal. No patient requires permanent discontinuation of SRL therapy. When dose reduction is necessary, amelioration of the toxic effects are evident within 24 hr, although up to 50 days are required for full recovery in a few patients [65].

## 4. Metabolic Disorders

The most common metabolic disorders following mTOR-I treatment are associated with a severe deregulation of the lipid and glucidic metabolism. Prevention, early recognition, and treatment of these complications may have a significant impact on long-term survival of these patients. 

### 4.1. Hyperlipidemia

Dyslipidemia is a major risk factor for posttransplant cardiovascular-related morbidity and mortality [69].

Several papers have reported that mTOR-I are well-recognized major causes of posttransplantation hyperlipidemia. They increase high-density lipoproteins (HDL), low-density lipoproteins (LDL), cholesterol, and triglycerides in approximately 40 to 75% of patients who receive this therapy [4, 70–72]. In the same way the upregulation of adipocyte fatty acid-binding protein (aP2) expressed in macrophages and monocytes plays a key role in the increased accumulation of triglycerides [73].

The mTOR-I-induced dyslipidemia constitutes a critical clinical problem because the annual risk of a cardiovascular event is almost 50-fold greater for a renal transplant patient than for the general population, and these events account for over one-third of all deaths [74].

A study of Morrisett et al. observed that cholesterol and triglyceride levels increase after 2–4 weeks of initiation of therapy, and this alteration reverted to near-baseline levels within 8 weeks after discontinuation of treatment. This demonstrated that hyperlipidemia is reversible and dose dependent [75].

The 3-Hydroxy-3-methyl coenzyme A (HMG-CoA) inhibitors (statins) alone or in combination with a second-line agent remain the main therapeutic option for mTOR-I-induced hyperlipidemia [76]. 

### 4.2. Posttransplantation Diabetes

Posttransplantation diabetes is a relatively frequent and unfortunate complication in patients carrying renal allografts [77]. All available information regarding potentially modifiable factors associated with or leading to diabetes should be part of a thoughtful decision-making process regarding the optimal maintenance of immunosuppression. 

To date, only a few clinical studies have suggested that SRL and its analogues are associated with hyperglycemia [78–81].

The mechanisms by which SRL may cause new-onset diabetes (NOD) are not clearly defined. SRL acts on the mTOR, a serine/threonine kinase that integrates signals from various nutrients and growth factors to regulate protein translation through a variety of downstream effectors. Physiologic conditions such as hyperinsulinemia promote serine/threonine phosphorylation of insulin receptor substrate proteins that inhibits their function and promotes their degradation. Overactivation of mTOR/S6K cascade would exert a significant negative effect on the activity of downstream components of the insulin/PI3-K pathway, such as AKT, leading to insulin resistance [82, 83]. Inhibitors of mTOR would therefore be expected to prevent development of insulin resistance through this mechanism. 

Additionally, Di Paolo et al. [82] studied 30 patients treated with long-term SRL and reported an unexpected impairment of insulin receptor substrate signaling and AKT activation, a finding that could help to explain deterioration of glucose metabolism in SRL-treated patients.

Other mechanisms that have been proposed for the induction of hyperglycemia by SRL include ectopic triglyceride deposition leading to insulin resistance [84, 85], impairment of insulin-mediated suppression of hepatic glucose production, or a direct toxic effect on pancreatic *β* cells [86, 87].

Another interesting hypothesis is that mTOR is involved in insulin signaling, and its inhibition may impede insulin-related gene transcription and expression, including glucose transporters. In particular, SRL abrogated the insulin-mediated increase in GLUT1 protein synthesis through partial inhibition of GLUT1 mRNA translation and partial inhibition of the rise in GLUT1 mRNA, resulting in the failure of insulin to stimulate glucose uptake. Similarly, mTOR is an inducer of ribosomal S6 kinase (S6K), and SRL blocks S6K activation or induces S6K inactivation through inhibition of T389 phosphorylation interfering with the transcript of insulin. This action may impact blood sugar levels [88].

Currently, it is considered that patients with an A1C assay ≥6.5% should start on glucose-lowering agents. As for type 2 diabetes, a stepwise approach should be adopted. The first step includes hygiene-dietetic recommendations (weight control, diet, and exercise). The second step is the initiation of an oral agent in monotherapy. The choice of the drug should take into account the patient-specific factors, graft function (some drugs or active metabolites are eliminated by the kidney), specific side effects, and potential pharmacokinetic interactions with immunosuppressive drugs (mainly interaction with CNI or mTOR-I through metabolism mediated by cytochrome P450, family 3, subfamily A, polypeptide 4/5 [CYP3A4/5]). The third step is a combination of oral agents with different mechanisms of actions. Combination therapy has not been investigated in kidney allograft recipients. The last step is the initiation of insulin with or without oral agents. If the target for glucose control is not achieved within 2–4 months, lifestyle interventions should be reassessed and patients should move to the next step [77].

About the immunosuppressive management of patients undergoing mTOR-I-related posttransplantation diabetes, there are still not well-defined guidelines, and although the switch to cyclosporine might be considered in selected patients, at the state of the art, no randomized clinical trial has been performed to better address this important point. 

Teutonico et al. then reported that the discontinuation of CNIs and their replacement by SRL fails to ameliorate the glycometabolic profile of kidney transplant recipients. Rather, it is associated with a worsening of insulin resistance and an inappropriately low insulin response [79].

 Unfortunately, experimental and clinical data on EVR are more scant.

### 4.3. Metabolic Syndrome

The concept of metabolic syndrome was first described by Reaven [89] as a combination of central obesity, dyslipidemia, hypertension, and fasting hyperglycemia, all thought to be based on insulin resistance and inflammation as the common pathophysiologic disturbances. In the general population, the presence of metabolic syndrome is associated with a risk for overt diabetes and cardiovascular diseases [90–92]. In addition, metabolic syndrome has been associated with proteinuria and reduced GFR, suggesting a link to chronic kidney disease [93, 94]. To the extent that diabetes (i.e., new-onset diabetes after transplantation), cardiovascular disease, and proteinuria are common complications of kidney transplantation, the role of metabolic syndrome in kidney transplantation recently has attracted a great deal of interest. Finally, the pathophysiology of the syndrome observed in the general population is dramatically altered by the effects of immunosuppressive medications in kidney transplant recipients. MTOR-I may have a primary effect [95]. 

## 5. Hypophosphatemia

A reduction in phosphate levels is an important toxicity of mTOR-I, therapy but the exact mechanism of this effect is not known. Symptoms of hypophosphatemia, including fatigue and weakness, occur when plasma phosphate concentration is <2 mg/dL [96], but when the levels drop to <1 mg/dL, serious complications can take place including confusion, weakness, myocardial dysfunction, and rhabdomyolysis [97]. 

Oral supplementation or the increment of phosphate intake from diet may be adequate for most patients.

## 6. Lymphedema

A study of Langer and Kahanshows that up to 38% of patients treated with cyclosporine and prednisone in association with SRL may present lymphoceles [98]. Lymphedema is a relatively rare adverse effect of mTOR-I therapy [99–102], but the underlying biological/physiological mechanism is not completely clarified. 

Aboujaoude et al. have hypothesized that the lymphedema could be strongly associated with the enhanced lymph flow and the lymphatic disruption secondary to the surgical procedures, together with the well-known increased vascular permeability and vasodilation caused by SRL [103]. 

Moreover Huber et al. showed an antilymphangiogenic activity of mTOR-I which is the common denominator in the pathophysiology of edematous states. In particular they reported that rapamycin administration impairs downstream signaling of VEGF-A through inhibition of the mTOR/p70S6K pathway in lymphatic endothelial cells (LECs) and also interferes with the intracellular pathway activation of LEC by VEGF-C, the main initiator of lymphangiogenesis. Interestingly this antilymphangiogenic activity is not restricted to a specific mTOR-I; rather it is a general phenomenon of mTOR inhibition [104]. 

Before establishing the diagnosis of lymphatic disease caused by mTOR-I, it is necessary to rule out other potential causes such as neoplasia, infection, venous obstruction, and genetic predisposition. A chest radiography, CT, or ultrasound study and laboratory tests can help in the differential diagnosis.

Reduction or discontinuation of the immunosuppressive drug therapy is, at the state of the art, the only worthy strategy in patients with lymphedema.

## 7. Cardiovascular Disease

The renal transplant population is highly vulnerable to premature CVD, the major cause of death with a functioning graft [105]. The risk of occurrence of a cardiovascular event in renal transplant patients treated with mTOR-I could be related to hypercholesterolemia, hypertriglyceridemia, and new-onset diabetes. However, to the best of our knowledge, at the state of the art, no reports (including the most important clinical trials) have strongly demonstrated that the presence of CV risk factors actually translates into an increased incidence of CV events or CV diseases. 

However, some *in vitro* experiments have also reported a prooxidant induced by mTOR-I whose effects may decrease NO availability [106–111].

This result is in line with some recent evidence reported by international cardiovascular research groups suggesting that SRL causes marked vascular dysfunction and nitrate resistance after continuous treatment for 7 days in animal model. This impaired vasorelaxation may, in part, be induced by upregulated mitochondrial superoxide release as well as by an upregulation of NADPH oxidase-driven superoxide production. Both processes could contribute to endothelial dysfunction observed after coronary vascular interventions with sirolimus-coated stents [111]. 

Another debated adverse effect related to mTOR-I is hypertension. As underlined by Reis et al. mTOR-I, similarly to CNI, have hypertensive effects. However, while CNI significantly promotes tachycardia and oxidative stress, mTOR-I seem to mainly interfere with lipid profile, hemorheology, and serotonin (5-HT) levels, without the same influence on catecholamine contents and lipid peroxidation. Thus, the cardiovascular disturbances underlying arterial hypertension development might be associated with distinct molecular/cellular signatures hypothetically explained by differences in the mechanism of action of immunosuppressants [112]. 

On the contrary, Joannidès et al. [113] have recently demonstrated that a CsA-free regimen based on SRL reduces aortic stiffness, plasma endothelin-1, and oxidative stress in renal recipients suggesting a protective effect on the arterial wall that may be translated into cardiovascular risk reduction. This study was in line with others demonstrating a lower hypertension development in mTOR-I-treated compared to CNI inhibitors-treated patients [114, 115]. 

However, based on contradictory research reports, it is unquestionable that these data are insufficient to define the mTOR-I systemic cardiovascular influence and to draw definitive clinical conclusions. Therefore, additional studies are necessary to evaluate short- and long-term CV effects of mTOR-I treatment in renal transplant patients. 

## 8. Cutaneous Adverse Effects

The clinical side effects of mTOR-I in the field of dermatology are edema, acne, epistaxis, aphthous ulceration, and vasculitis. Mahé et al. have performed a cross-sectional study in renal transplant recipients receiving SRL which underwent a dermatological examination. The main cutaneous adverse events they observed were infection, edema, mucous membrane pathologies, and nail disorders [116].

### 8.1. Acne

 Acne is reported in 15% to 25% of organ transplant recipients treated with SRL [117, 118]. Mahé et al. have reported skin eruptions resembling acne, mainly located on the face and trunk, in 46% of evaluated patients. Moreover the male predominance of these acne-like eruptions suggests that this side effect is hormone dependent [116].

### 8.2. Mucous Membrane Disorders

One of the most common side effects reported in clinical trials of mTOR-I has been mucositis, probably because of the direct toxic effect of these drugs on oral and nasal mucous membranes. Numerous clinical trials have reported aphthous ulcerations or oral ulcerations [47, 119]. Frequently they are confined to the soft mucosa of the mouth and very common on the tongue and lips. Mouth ulcers usually occur just after the introduction of SRL treatment but most of the time are transient. However if persistent, these painful and debilitating lesions lead to either dose reduction or discontinuation of mTOR-I in a significant number of patients [120].

Topical steroid, iodine, or topical analgesic may be used to reduce pain and to promote disappearance of the ulcer. If symptoms persist, mTOR-I should be discontinued and possibly restarted at a lower dose after resolution of symptoms. Anyway it is important to educate patients to maintain good oral hygiene and inform patients about this side effect. Finally, it is important to perform a careful oral examination during the routine followup.

### 8.3. Edema

Chronic edema has been reported in 8% to 62% of renal transplant recipients receiving SRL treatment [103, 121, 122].

Mahé et al. have found chronic edemas (lasting more than 1 month, resistant to diuretics and without local, renal, or cardiac causes) in 55% of evaluated renal transplant recipients. Edemas affected primary lower limbs and are soft and noninflammatory. Angioedema (acute subcutaneous edema), found in 15% of patients, developed within a few hours and disappeared in less than 4 days. They were nonpruritic, nonerythematous, and localized mainly on the face, with oral cavity involvement [116]. 

### 8.4. Nail and Hair Pathologies

Nail abnormalities associated with SRL treatment include fragile and thin nails, longitudinal ridging, distal onycholysis, and erythema.

Skin and scalp hair abnormalities comprised mild alopecia or hypertrichosis of the face [116]. In most cases these adverse events were not serious, but in 12% of patients it has been necessary to withdraw the therapy. 

## 9. Gonadal Impact

In the last years several studies have emphasized the impact of SRL on male gonadal function. In detail these studies have focused on sex hormones production, erectile function, and fertility. Regarding the balance on sex hormones production, three studies have revealed significantly lower testosterone levels and a significant increase in gonadotrophic hormones (FSH and LH) in patients treated with SRL [123, 124]. As regard the impact of SRL on sperm, a significant reduction in total sperm count and fathered pregnancy rate in patients who receive SRL compared to patients with SRL-free regimen has been shown [125, 126]. The molecular mechanisms by which mTOR-I induces a decrease in testosterone level and sperm impairment still remain partly unknown. In an animal model Feng et al. found that SRL plays a central inhibitory role on a stem cell factor (SCF)/c-kit-dependant process in spermatogonial proliferation via the PI3-K/AKT/p70S6K pathway [127].

## 10. Bone Diseases

Posttransplant renal osteopathy is a clinical posttransplant complication associated with morbidity and mortality due to the increased frequency of bone fractures compared with general population [128]. Osteopenia, osteoporosis, and osteonecrosis represent the most common complications related to renal transplant [129]. In a cross-sectional study, osteoporosis was observed in 40% and bone fracture in 44% of renal transplant recipients evaluated 8 years after transplantation [130]. Certainly, steroid treatment represents the more important pathogenic factor of osteopenic-osteoporosis syndrome in these patients. In experimental study on rats, SRL increased remodeling and growth retardation of bone but did not produce bone loss [131]. 

Contrarily, EVR directly inhibits bone resorption controlled by osteoclasts, and then it should be used in patients with concomitants bone disease [132]. 

## 11. Surgical Wound Complication

SRL treatment has been reported to be associated with a greater incidence of wound-healing problems than other maintenance immunosuppressive agents [133–136]. This effect is likely related to its ability to impair signal transduction of fibroblast and endothelial growth factors [137].

Knight et al. have evaluated the risk factors for wound complications (infection, lymphocele, and/or incisional hernia) in renal transplant recipients with de novo SRL treatment. They found that one-third of recipients suffered at least one wound complication after one-year followup. The independent risk factors for the development of these complications are age, obesity, Caucasian race, and high dose of SRL in the first few days after transplantation. They concluded with useful recommendations including the avoidance of a large loading dose of SRL and delay the introduction of this agent for several days particularly for Caucasian, older aged recipients, and patients with a BMI > 30 [138]. 

## 12. Infections

The severe infections are frequent cause of mTOR-I discontinuation [18, 139, 140] and one of the leading causes of death in renal transplant recipients [141, 142]. In particular, several biomolecular studies have shown that SRL is able to inhibit interleukin-12-induced proliferation of activated T lymphocytes [143] and IFN-*γ* production of the lymphocytes. Both cytokines are known to be critical in protective immunity to intracellular bacteria (e.g., mycobacterium) [144]. 

Literature evidence, then, suggests that both SRL- and EVR-based regimens are associated with low CMV infection rate in comparison with other immunosuppressive regimens [141, 145]. The mechanism underlying the apparent beneficial effect of SRL in reducing the risk of CMV-related infection and invasive disease in this patients' population remains speculative but may relate to the effect of mTOR inhibition on CMV replication. As an obligate intracellular organism, CMV must utilize the intrinsic metabolic pathways of the host cell to direct the synthesis of proteins that are essential to its replication. Since mTOR serves as a key regulator of cellular protein synthesis, its inhibition by SRL may, in turn, inhibit CMV replication. Experimental models of CMV infection suggest that SRL impairs, but does not entirely prevent, CMV replication, so other mechanisms are likely involved as well [146].

However, further investigations should be undertaken to better analyze the biological/biomolecular machinery associated with this condition, and randomized clinical trials with homogeneous antiviral prophylaxis, standardized definitions, and adequate statistical power need to be performed to confirm these clinical observations.

## 13. Others

Other complications, reported in approximately 15–20%, include gastrointestinal side effects (e.g., diarrhea, vomiting, and anorexia) [147]. Gastrointestinal leukocytoclastic vasculitis is a rare adverse effect reported in a few cases of use of SRL. It is characterized by diffuse mucosal thickening of the antrum, duodenum, and jejunum. Esophagogastroduodenoscopy revealed erythema, swelling, and white plaques in the antrum, asymmetrical pylorus, and granular swelling of the third portion of the duodenum [148]. In presence of these complications, drug discontinuation is encouraged. 

Fatigue, alterations in taste, and asthenia are other common toxicities. These symptoms were usually manageable with a reduction in the drug dose [149]. 

## Figures and Tables

**Table 1:** Most common adverse events in mTOR-I-treated renal transplant recipients.

Adverse events	Rate of occurrence (%)	References
Pulmonary toxicity	2–11	[20, 21, 24, 33]
Hematopoietic adverse effects		
Anemia	13–58	[6, 36, 44–47, 50, 56, 57, 70, 72, 135, 147]
Leukopenia	5–39	[6, 45, 46, 56, 66, 117, 121, 147]
Thrombocytopenia	4–45	[6, 45–47, 56, 66, 70, 117, 118, 121, 122, 147]
Metabolic disorders		
Hyperlipidemia	8–87	[6, 45–47, 57, 66, 70–72, 115, 117, 118, 121, 135, 147]
Posttransplantation diabetes	3–33	[56, 70, 72, 78, 80, 115, 121, 138, 147]
Hypophosphatemia	15–20	[45, 46, 57]
Lymphedema	<5	[99–102]
Cardiovascular disease	1–6	[80, 100, 117, 122, 124, 128]
Hypertension	8–58	[46, 57, 70, 72, 115, 117, 121, 122, 135]
Cutaneous adverse effects		
Acne, folliculitis	9–25	[6, 57, 70, 116–118, 135, 147]
Stomatitis and mucous membrane disorders	9–64	[6, 118, 138, 147]
Edema	2–70	[6, 56, 57, 70, 121, 122, 135, 147]
Nail and hair pathologies	74	[116]
Gonadal complications	<5	[123–126]
Surgical wound complication	2–20	[56, 70, 72, 133–136]
Infections	2–60	[6, 72, 117, 122, 136]
Gastrointestinal complication	2–51	[6, 46, 47, 56, 57, 70, 72, 117, 118, 121, 135, 147]
